# Use of Modern Family Planning Methods in Fishing Communities of Lake Victoria, Uganda

**DOI:** 10.1371/journal.pone.0141531

**Published:** 2015-10-29

**Authors:** Annet Nanvubya, Julius Ssempiira, Juliet Mpendo, Ali Ssetaala, Annet Nalutaaya, Mathias Wambuzi, Paul Kitandwe, Bernard S. Bagaya, Sabrina Welsh, Stephen Asiimwe, Leslie Nielsen, Fredrick Makumbi, Noah Kiwanuka

**Affiliations:** 1 UVRI-IAVI HIV Vaccine Program, Entebbe, Uganda; 2 Kabwohe Clinical Research Center (KCRC), Kabwohe, Uganda; 3 International AIDS Vaccine Initiative, New York, United States of America; 4 Makerere University College of Health Sciences, School of Public Health, Kampala, Uganda; Indiana University, UNITED STATES

## Abstract

**Introduction:**

Fishing communities (FCs) in Uganda have high HIV infection rates but poor access to health services including family planning (FP). Although FP is a cost-effective public health intervention, there is a paucity of data on knowledge and use of modern FP in FCs. This study determined knowledge and use of modern FP methods in FCs of Uganda.

**Methods:**

Data were accrued from a 12-month follow up of 1,688 HIV-uninfected individuals, 18–49 years from 8 FCs along Lake Victoria, between September 2011 and March 2013. Data on knowledge and use of modern FP were collected through a semi-structured questionnaire. Prevalence Risk Ratios with corresponding 95% CIs were used to determine factors associated with Modern FP knowledge and use.

**Results:**

The mean age was 31.4 years, with nearly half (48.8%) being females while more than half (58.6%) had attained up to primary education level. Knowledge of modern FP was high, 87.5% (1477/1688); significantly higher among females [adj. PRR = 4.84 (95% CI; 3.08, 7.61)], among older respondents (25–29 years) [adj. PRR = 1.83 (95% CI; 1.12, 2.99)] compared to younger ones (18–24 years) and among those conducting business [adj. PRR = 2.42(95% CI; 1.02, 5.74)] relative to those primarily in fishing. Just over a third (35.2%, 595/1688) reported use of at least one modern FP method. Use of modern FP methods was significantly higher among females [adj. PRR = 2.04 (95% CI; 1.56, 2.65, and among those reporting multiple sexual partnerships [adj. PRR = 2.12, 95% CI; 1.63, 2.76)]. Nonuse of modern methods was mostly due to desire for more children (30.6%), fear of side effects (12.2%) and partner refusal (5.2%).

**Conclusion:**

Despite their high knowledge of FP, FCs have low use of modern FP methods. Key barriers to use of modern FP methods were high fertility desires, fear of perceived side effects and partner refusal of methods.

## Introduction

Fishing communities(FCs) are considered among the most at risk populations (MARPS) because of their high vulnerability to HIV infection and other sexually transmitted infections (STIs)[1–3]. FCs in low- or middle-income countries in Africa, Asia and Latin America have HIV prevalence rates that are five to ten times higher than that of the general population[4]. In Uganda, FCs have been shown to have HIV infection rates approximately three to six times higher than in the general population [1,5–9]. This vulnerability stems from complex, interdependent causes which include the mobility of many fisher folk, the time fishermen and fish traders spend away from home, their access to daily cash income in an overall context of poverty and vulnerability, their demographic profile (they are often young and sexually active) and the ready availability of commercial sex in many fishing villages [5–7,10]. There is a high level of sexual mixing and relatively low level of condom use within these communities which makes it very likely that STIs, including HIV, will spread rapidly to all sexually active individuals[11]. Because of the high HIV rates, these communities are being targeted for HIV care and prevention interventions which include use of modern Family Planning (FP) methods. Modern methods of FP include female and male sterilization, oral hormonal contraceptives, intra-uterine devices (IUD), male and female condoms, injectable contraceptives such as Depo-provera, hormonal implants (including Norplant) and emergency contraception. FP is needed to postpone pregnancy, prevent HIV transmission to other sexual partners (through condom use) and in the Elimination of Mother- to- Child Transmission (EMTCT) of HIV infection. It is also crucial in the prevention of other sexually transmitted diseases (STIs) which are predisposing factors in the acquisition of HIV. FCs in Uganda are socially marginalized and have extremely limited access to basic healthcare services including modern FP[11,12], a finding that makes these communities different from others. Given its public health importance, the lack of modern FP may be another contributing factor to the high HIV infection rates in these FCs.

Most developing countries have a higher unmet need for modern FP as compared to that in developed countries [13]. By 2013 the unmet need for FP in most sub-Saharan countries remained between 13–34% and yet by 2010 the unmet need for FP in married women in the United States of America was only 8%[14]. Uganda has an unmet need for modern FP estimated at 34.5% and a total fertility rate of approximately seven children per woman of reproductive age [10]. There is currently no data on use of modern FP by people living in FCs of Uganda. Because of their high HIV infection rates, the integration of FP services into HIV prevention and care services is a worthwhile intervention to consider. Against this background, we explored the knowledge and use of modern FP methods in FCs of Lake Victoria in Uganda with the aim of understanding their reproductive health needs. Data accrued from this research will inform service provision strategies in these communities, HIV prevention campaigns and the design and integration of FP services in future research interventions.

## Materials and Methods

### Study setting and population

Eight FCs (one landing site and 7 Islands) in three Ugandan administrative districts of Wakiso, Mukono and Kalangala were selected based on geographical location, size and population. The studied FCs included Kasenyi landing site and Islands of Myende, Namisoke, Kiimi, Makusa, Jaana, Kavenyanja and Zinga. These communities are remotely located and face challenges with regard to land and water transport. Uganda is a multi-ethnic country with the Baganda as the majority. Other ethnic groups include the Banyankore, Basoga, Banyoro, Batoro, Bagisu, Luo and are collectively referred to in this paper as non-Baganda. This study was conducted in an area predominately inhabited by the Baganda ethnicity.

A community household census was performed between September 2011 and March 2013, followed by a demographic baseline socio-behavioral survey conducted amongst 2,200 HIV uninfected respondents aged 18 to 49 years as previously described[8]. The study area had a combined approximate population of 36,000 people with Kasenyi landing site having the largest population of approximately 10,000 people while the island communities had populations ranging from 1,000 to 7,000 people. The entire study population is served by three government health facilities often with few qualified stationary health personnel. It is also infrequently served by non-government mobile health/research organizations such as the Uganda Virus Research Institute (UVRI) -International AIDS Vaccine Initiative (IAVI), Uganda Health Marketing Group (UHMG), Entebbe hospital and Marie Stopes; the later focuses on FP.

At the 12-months study follow-up visit, knowledge and use of modern FP methods were assessed using a semi-structured, face-to-face interviewer administered questionnaire among 1,688 consenting adults. Data collected included knowledge of any FP method, use of any FP method either by the respondent or their partner, sources of methods, perceived effectiveness and reasons for non-use. Knowledge of modern FP method was defined as being aware of any modern FP method and knowing how it works. Non-users in this study were defined as those respondents who did not use any modern FP method as defined above. Data were collected by trained male and female community data collectors, edited and quality controlled in real time.

### Ethics statement

The study was approved by the Uganda Virus Research Institute, Science and Ethics Committee (UVRI-SEC, FWA number 00001354) and the responsible regulatory authority, Uganda National Council of Science and Technology (UNCST, FWA number 00001293). Written informed consent was obtained from all respondents prior interviews. Condoms were given to all participants and anyone who requested them. Those diagnosed with HIV infection were counseled and referred to local service providers for further care and management. Those diagnosed with syphilis and other symptomatic STIs were counseled and offered treatment with their partners.

### Data management and statistical analyses

Data were captured by two independent data entry clerks using the EPI-DATA software with range, consistence and logic checks. Respondent characteristics were described using proportions for categorical data and mean or median for continuous data. Differences in characteristics were assessed using chi-square and Fisher's exact tests for categorical variables and Student *t-* tests for continuous variables. The primary outcome variables were knowledge of modern FP methods and modern FP use, both coded as categories (1- if knowledgeable on/using any modern FP method, and 0- if not). Factors associated with knowledge and use of modern FP were determined using prevalence risk ratios (PRR) with corresponding 95% Confidence intervals (CIs) as the measure of association estimated through use of a log-binomial generalized linear model. Variables for inclusion in multivariable analysis were selected on the basis of a p- value of <0.1 at bivariate analysis, biological plausibility, potential confounders and known factors from prior studies[15,16]. The final models were adjusted for gender, age, education, marital status, occupation, religion and ethnicity. In addition, the FP use final model was adjusted for district of residence and having other sexual partner(s) at the time of interview. Statistical analyses were performed using Stata^®^ 13 (StataCorp, college Station, TX, USA) software.

## Results

### Study population

The socio-demographic characteristics of the 1,688 respondents who were followed up are shown in Table 1. Almost one half (48.8%) of the respondents were female, engaging in fishing or related activities such as fish processing and boat building. The majority of respondents had completed primary school, were married and non-Baganda. Approximately a quarter of the respondents reported having multiple sexual partnerships.

**Table 1:** Socio-demographic characteristics of the respondents by gender (N = 1688).

Characteristic	Total number (%)	Males (%)	Females (%)
	1688	864 (51.2)	824 (48.8)
Mean	31.4	32.1	30.6
Standard Deviation (SD)	7.5	7.5	7.4
Interquartile Range (IQR)	26–37	26–38	25–35
Median	30	31	30
**Age at enrolment (years)**			
18–24	323 (19.1)	142 (16.4)	181 (22.0)
25–29	445 (26.4)	219 (25.4)	226 (27.4)
30–34	380 (22.5)	189 (21.9)	191 (23.2)
35–39	264 (15.6)	154 (17.8)	110 (13.4)
40–49	264 (15.6)	160 (18.5)	116 (14.1)
**Highest education level**			
None	138 (8.2)	59 (6.8)	79 (9.6)
Primary	990 (58.6)	498 (57.6)	492 (59.7)
Secondary and above	560 (33.2)	307 (35.5)	253 (30.7)
**Religion**			
Roman Catholic	682 (40.4)	350 (40.5)	332 (40.3)
Protestant/Anglican	453 (26.8)	237 (27.4)	216 (26.2)
Moslem	331 (19.6)	182 (21.1)	149 (18.1)
Pentecostal	160 (9.5)	64 (7.4)	96 (11.7)
Other*	62 (3.7)	32 (3.7)	30 (3.6)
**Ethnicity**			
Non-Baganda	884 (52.4)	475 (55.0)	409 (49.6)
Baganda	804 (47.6)	389 (45.0)	415 (50.4)
**Occupation**			
Fishing/fishing related	845 (50.1)	640 (74.1)	205 (24.9)
Trade / business	168 (10.0)	48 (5.6)	120 (14.6)
Bar / lodge	163 (9.7)	9 (1.0)	154 (18.7)
Farming	136 (8.1)	55 (6.4)	81 (9.8)
Housewife	134 (7.9)	0 (0)	134 (16.3)
Other¶	242 (14.3)	112 (13.0)	130 (15.8)
**Marital status**			
Married	1147 (67.9)	589 (68.2)	558 (67.7)
Never married	373 (22.1)	205 (23.7)	168 (20.4)
Separated or divorced	68 (4.0)	70 (8.1)	98 (11.9)
**Having other sexual partner(s) in addition to spouse**			
No	1249 (74.0)	573 (66.3)	676 (82.0)
Yes	439 (26.0)	291 (33.7)	148 (18.0)
**District of Residence**			
Wakiso	837 (49.6)	410 (47.5)	427 (51.8)
Mukono	361 (21.4)	185 (21.4)	176 (21.4)
Kalangala	386 (22.9)	226 (26.2)	160 (19.4)
Other	104 (6.2)	43 (5.0)	61 (7.4)

*Seventh Day Advent/Traditionist,

^¶^Construction/Mechanic/Government/Clerical, Δ Luweero /Mubende.

### Knowledge of family planning

Participants’ knowledge of modern FP is shown in Table 2. Knowledge of modern FP was defined as being aware of any modern FP method and knowing how it works. Approximately 88%of the respondents knew about modern FP methods, with women and older respondents having more knowledge (P<0.01). At Multivariate analysis, knowledge of modern FP was significantly higher among females as compared to males, among older respondents aged 25 to29 years compared to their younger counterparts aged 18 to 24 years] and among the Baganda as compared to the non-Baganda (P<0.05). FP knowledge was 2.42 times higher for those in trade or business and 2.48 times higher in those working in bars or lodges relative to those who were primarily in fishing or other related activities. Respondents with a higher level of education and those who were single (never married, separated or divorced) were more likely to have knowledge about modern FP methods (P<0.05). There was no significant difference in knowledge of modern FP across different religious denominations.

**Table 2:** Unadjusted and adjusted Prevalence Risk Ratios (PRRs) of factors associated with modern FP knowledge (N = 1688).

Characteristic	FP Knowledge	Unadjusted PRRs	Adjusted PRRs	P-value
	% (n/N)	(95% CI)	(95% CI)	
Reported FP knowledge	87.5 (1477/1688)			
**Gender**				
Male	80.1 (692/864)	1		
Female	95.3 (785/824)	5.00 (3.48, 7.19)	4.84 (3.08, 7.61)	<0.001††
**Age (years)**				
18–24	85.7 (277/323)	1		
25–29	91.5 (407/445)	1.78 (1.13, 2.81)	1.83 (1.12, 2.99)	0.016††
30–34	87.6 (333/380)	1.18 (0.76, 1.82)	1.09 (0.67, 1.76)	0.734
35–39	85.6 (226/264)	0.99 (0.62, 1.57)	1.08 (0.65, 1.80)	0.76
40–49	84.8 (234/276)	0.93 (0.59, 1.46)	1.06 (0.64, 1.74)	0.816
**Highest education level**				
None	76.1 (105/138)	1		
Primary	87.9 (870/990)	2.28 (1.47, 3.52)	2.94 (1.81, 4.78)	<0.001††
Secondary and above	88.2 (494/560)	2.35 (1.47, 3.76)	2.85 (1.68, 4.82)	<0.001††
**Religion**				
Roman Catholic	86.8 (592/682)	1		
Protestant/Anglican	88.1 (399/453)	1.12 (0.78, 1.61)	1.17 (0.79, 1.71)	0.437
Moslem	85.5 (283/331)	0.89 (0.61, 1.31)	0.89 (0.60, 1.33)	0.575
Pentecostal	91.9 (147/160)	1.72 (0.94, 3.16)	1.61 (0.85, 3.05)	0.147
Other*	90.3 (56/62)	1.42 (0.59, 3.39)	1.24 (0.49, 3.13)	0.655
**Ethnicity**				
Non-Baganda	85.2 (753/884)	1		
Baganda	90.0 (724/804)	1.57 (1.17, 2.12)	1.40 (1.02, 1.93)	0.039††
**Occupation**				
Fishing/fishing related	83.2 (703/845)	1		
Trade/business	96.4 (162/168)	5.45 (2.37, 12.56)	2.42 (1.02, 5.74)	0.046††
Bar/lodge	96.9 (158/163)	6.38 (2.57, 15.83)	2.48 (0.93, 6.61)	0.070†
Farming	86.0 (117/136)	1.24 (0.74, 2.09)	0.74 (0.42, 1.30)	0.294
Housewife	94.0 (126/134)	3.18 (1.52, 6.65)	0.67 (0.28, 1.60)	0.362
Other¶	87.2 (211/242)	1.37 (0.91, 2.09)	1.01 (0.64, 1.59)	0.953
**Marital status**				
Married	90.4(1037/1147)	1		
Never married	79.6 (297/373)	0.41 (0.30, 0.57)	0.39 (0.28, 0.57)	<0.001††
Separated or divorced	85.1 (143/168)	0.64 (0.38, 0.97)	0.48 (0.29, 0.80)	0.004††

* Seventh Day Advent/Traditionist,

^¶^ Construction/Mechanic/Government/Clerical,

^††^ statistically significant at p<0.05,

^†^ Borderline significant at p<0.10.

### Modern family planning use

Modern FP use by a respondent or their sexual partner was reported in just over a third (35.2%) of the respondents with more females than males reporting use as shown in Table 3. Short-acting reversible contraceptive methods such as oral contraceptives and Depo-Provera were used by three quarters of the respondents as compared to one quarter who used the long-acting reversible methods such as intra-uterine devices and implant. More than half of the respondents who reported knowing about modern FP methods were not using them (59.7%). FP use increased with education level, with the lowest use of FP reported by those without any formal education and the highest use by those who had attained secondary or higher level of education Muslims were less commonly using modern FP methods despite having knowledge. Those who were never married and those who were separated or divorced were more likely to use modern FP compared to those who were married (p<0.001). Modern FP use was 2.12 times higher among those who had more than one sexual partner compared to those who were monogamous (p<0.001). In the final model after multivariable analyses, age, ethnicity, occupation and district of residence lost statistical significance.

**Table 3:** Unadjusted and adjusted Prevalence Risk Ratios (PRRs) of factors associated with modern FP use (N = 1688).

Characteristic	Modern FP Use	Unadjusted PRRs	Adjusted PRRs	P-value
	% (n/N)	(95% CI)	(95% CI)	
Reported Use	35.3 (595/1688)			
**Gender**				
Male	29.2 (252/864)	1 (ref)		
Female	41.6(343/824)	1.73 (1.42, 2.12)	2.04 (1.57, 2.65)	<0.001††
**Age (years)**				
18–24	34.4 (111/323)	1 (ref)		
25–29	37.5 (167/445)	1.14 (0.85, 1.55)	1.10 (0.80, 1.51)	0.564
30–34	37.1 (141/380)	1.13 (0.83, 1.54)	1.11 (0.79, 1.55)	0.547
35–39	34.8 (92/264)	1.02 (0.73, 1.44)	1.18 (0.81, 1.70)	0.388
40–49	30.4 (84/276)	0.84 (0.59, 1.18)	1.00 (0.68, 1.46)	0.992
**Highest education level**				
None	31.2 (43/138)	1 (ref)		
Primary	33.5 (332/990)	1.11 (0.76, 1.64)	1.10 (0.74, 1.66)	0.619
Secondary and above	39.1 (220/560)	1.42 (0.96, 2.13)	1.47 (0.96, 2.25)	0.075†
**Religion**				
Roman Catholic	37.5 (256/682)	1 (ref)		
Protestant/Anglican	39.3 (178/453)	1.08 (0.84, 1.37)	1.04 (0.80, 1.34)	0.776
Moslem	27.8 (92/331)	0.64 (0.48, 0.85)	0.63 (0.47, 0.85)	0.002††
Pentecostal	30.0 (48/160)	0.70 (0.49, 1.03)	0.63 (0.43, 0.94)	0.023††
Other*	33.9 (21/62)	0.85 (0.49, 1.47)	0.80 (0.45, 1.41)	0.44
**Ethnicity**				
Non-Baganda	34.4 (304/884)			
Baganda	36.2 (291/804)	1.08 (0.89, 1.32)	1.01 (0.81, 1.24)	0.955
**Occupation**				
Fishing / fishing related	31.2 (264/845)	1 (ref)		
Trade / business	42.3 (71/168)	1.35 (1.04, 1.76)	1.21 (0.83, 1.76)	0.323
Bar / lodge	39.3 (64/163)	1.26 (0.96, 1.65)	1.04 (0.69, 1.55)	0.857
Farming	33.8 (46/136)	1.08 (0.79, 1.48)	0.98 (0.64, 1.50)	0.936
Housewife	44.8 (60/134)	1.43 (1.08, 1.90)	1.00 (0.65, 1.55)	0.984
Other¶	37.2 (90/242)	1.19 (0.94, 1.51)	1.20 (0.86, 1.66)	0.28
**Marital status**				
Married	40.7 (467/1147)	1 (ref)		
Never married	21.4 (80/373)	0.40 (0.30, 0.52)	0.29 (0.21, 0.39)	<0.001††
Separated or divorced	28.6 (48/168)	0.58 (0.41, 0.83)	0.43 (0.30, 0.63)	<0.001††
**Currently having other sexual partner(s)**				
No	33.8(422/1249)	1 (ref)		
Yes	39.4(173/439)	1.27 (1.02, 1.60)	2.12(1.63, 2.76)	<0.001††
**District of Residence**				
Wakiso	35.2(295/837)	1 (ref)		
Mukono	35.5(128/361)	1.09 (0.78, 1.31)	1.01 (0.77, 1.33)	0.936
Kalangala	34.5(133/386)	0.97 (0.75, 1.24)	1.02 (0.78, 1.34)	0.862
OtherΔ	37.5(39/104)	1.10 (0.72, 1.68)	1.04 (0.66, 1.64)	0.853

* Seventh Day Advent/Traditionist,

^¶^ Construction/Mechanic/Government/Clerical

^Δ^ Luweero /Mubende,

^††^ statistically significant at p<0.05,

^†^ Borderline significant at p<0.10.

### Reasons for non-use of modern FP

All respondents who were not using FP (65.0%) were asked to give the reasons why they or their partners were not using FP. About one third (30.6%) were not using FP because they desired to have more children, 12.2% feared side effects, while 8.7% reported partners refusing to allow them to use modern FP as shown in Table 4. Twenty seven percent were not in a sexual relationship at the time of interview while 7.8 percent were pregnant or breast feeding. More females than males reported limited access of FP (88.9% vs. 11.1%). Only eight respondents cited religious reasons for non-use while thirty one believed that FP was not effective. Out of the five who cited traditional or tribal cultural demands to have many children, four were male.

**Table 4:** Reasons for non-use of modern FP (N = 1093).

Reason for not using modern FP	Total number (%)	Male (%)	Female (%)
	1093	438 (40.1%)	655 (59.9%)
I want to have more children	334 (30.6%)	112 (33.5%)	222 (66.5%)
Currently not in a sexual Relationship	295 (27.0%)	167 (56.6%)	128 (43.4%)
FP methods have side effects	133 (12.2%)	33 (24.8%)	100 (75.2%)
Currently Pregnant/Breastfeeding	85 (7.8%)	23 (27.1%)	62 (72.9%)
I am still young (Not yet ready to use Modern FP)	78 (7.1%)	36 (46.2%)	42 (53.8%)
Husband/partner refusal of FP	8.7% (57/655)	0	57 (100%)
Only married for a few years	57 (5.2%)	16 (28.1%)	41 (71.9%)
Not being fertile/Post-menopausal	44 (4.0%)	4 (9.1%)	40 (90.9%)
FP methods are not effective	31 (2.8%)	8 (25.8%)	23 (74.2%)
Limited access of FP methods	18 (1.7%)	2 (11.1%)	16 (88.9%)
Not wanting to use FP	15 (1.4%)	8 (53.3%)	7 (46.7%)
Religion does not permit use of FP methods	8 (0.7%)	3 (37.5%)	5 (62.5%)
Culture encourages having many children	5 (0.5%)	4 (80.0%)	1 (20.0%)
Other	70 (6.4%)	38 (54.3%)	32 (45.7%)

## Discussion

Our findings show that although the people living in FCs of Lake Victoria in Uganda have a high level of knowledge about the concept of FP, their use of modern FP is low. This is consistent with what has been observed in the general population[17,18]. This trend is also similar to what has been observed in other African countries where high FP knowledge does not translate into use [19–22]. Contrary to what is observed in this study population and elsewhere in Africa, both knowledge and use of modern FP in many developed countries are high [1]. Perhaps, more research is needed to further understand factors that contribute to low usage of modern FP despite high knowledge in African settings.

In this study, women respondents and those who had attained a higher education level were more likely to know about FP. In a study about ethnic women in aquaculture in Nepal, it was noted that most ethnic minority populations are less aware about FP due to less access to education and low participation in social activities[23]. Findings from a study which looked at trends and determinants of contraceptive use in Rakai district of Uganda also showed that women and respondents who are highly educated were more likely to know about modern FP[16]. Extensive campaigns for modern FP have made a positive contribution toward public awareness of contraceptive methods country-wide, however it is possible that sexual and reproductive health services mostly target women and youth as these tend to be sexually vulnerable. Concerted efforts to increase male involvement in sexual and reproductive health issues have been promoted as male involvement is reported to positively affect contraceptive use[24,25]. Within communities where men are the decision-makers for health issues and the application of resources, involving them in sexual and reproductive health issues could encourage them to make valuable decisions which benefit their families as a whole [24]. This involvement ought to be at the concept level, the ‘supportive’ level (being supportive for spouses to use contraception) and the ‘acceptor’ level (as FP users). It has been previously demonstrated in recent studies that the majority of people in FCs do not get formal education or if they do, very few go up to tertiary education level[5,8]. Because FCs are remote, they tend to be deprived of social services like formal education which may impact their uptake of health services [3,4]. Whereas modern FP knowledge and use increased with education level, we found that only one third of the study population had attained secondary or higher education level. Previous research in a similar setting also revealed that higher levels of education were associated with higher levels of contraceptive FP knowledge and use[16]. This highlights the significant role education plays in the uptake of health services including sexual and reproductive services. Social services need to be prioritized in FCs as a strategy to reduce their high HIV infection rates.

While it is widely accepted that FCs are occupied by only fishermen, these communities are inhabited by people with various occupations that include fishing, farming, trade or business and others. Our findings show that modern FP knowledge is higher in traders or business people and those working in bars or lodges as compared to those involved primarily in fishing or fishing-related jobs. These subgroups have been previously reported to have more money than others living in the same community[6]. Because of this and the mobile nature of their work, business people and bar attendants tend to be involved in commercial or multiple sexual relationships which may cause them to seek out contraceptive knowledge and to have a profound need for contraception. It is not surprising that those who were having other sexual partners were shown to be more likely to use modern FP as compared to those who did not have them.

FCs in Uganda are amongst population groups that are most at risk of HIV infection, which makes use of modern FP in these communities particularly critical[5,8]. Due to their remoteness and geographical location, fisher-folk communities are often hard to reach [26]. They normally have weak health systems as exemplified by stock outs of medical supplies, insufficient staffing or inadequately trained staff [5]. This might explain the low contraceptive prevalence rate in this study population.

Different reasons for not using FP have been reported in many studies from different settings [16,21,27]. The most common reasons cited by respondents in this study population for not using modern FP included, fertility desires (wanting to have many children) and, fear of side effects such as excessive bleeding following use of hormonal contraceptive methods. The desire to have many children has been reported before as a major hindrance to the use of modern FP [16,28]. Having many children impacts a family’s wellbeing as it depletes its resources and eventually impacts a country’s economic growth [29]. Programs aimed at increasing modern FP use should sensitize the public about the dangers of having a large family and the impact this has on families and the nation at large. Elsewhere, sensitization on the management of side effects of the different FP methods has helped to allay unnecessary anxieties that people have about FP [12]. It is however important to note that some of the side effects in this population were rather presumed or anticipated as has been reported by other researchers[12,24]. FP messages should be tailor made to address the concerns of different populations.

Religion and ethnicity are critical sociocultural dimensions that have for long been considered as having important influences on attitudes towards, and the uptake of FP methods [30]Moslems and Pentecostals in this study population were less commonly using modern FP despite knowledge. Religious leaders ought to be sensitized about benefits of FP and encouraged to promote use of FP by their followers.

Support for regulation of individual fertility has been evident in all cultures, and at all times, even in those societies in which social and religious rules have favoured production of children [31]. In some African countries, people attach prestige to having many children which may explain the low utilization of modern FP in this study population [17]. Some women in developing countries have limited ability to make an independent decision to use medical services. This is evidenced in this study where fifty seven women cited husband or partner refusal to use FP. It has been shown that the decision-making role of husbands/partners has to be taken into account while packaging counseling messages in order to promote FP use[24,25].

Whereas the majority of the respondents reported that they considered their FP method choice effective, there are some who believed that their method choice was not effective. This highlights a knowledge gap in regard to mechanism of action of the different FP methods. Raising awareness on mode of action, eligibility criteria, advantages and side effects of the different modern FP methods has been found to be a great remedy for this gap by some researchers [25]. Nevertheless, if modern FP methods are not used correctly, their effectiveness is compromised.

Limited access to FP services was reported by some of those who were not using modern FP. Some of the reasons cited for limited access included long distances to mainland centers where FP services are available while others included lack of transportation to these centers and money to pay for the FP services. It is logical that in these hard-to reach communities, people will go to health facilities or for services on the basis of proximity and awareness of the services available. If reproductive services are not brought closer to the people who need them, then the people will find it hard to access them.

Other factors which hindered respondents from using FP included a prevailing belief that contraception is a woman's concern, a belief that has been reported in other studies from other countries[21]. The fact that most modern FP methods are for women tends to make people think that FP is a woman’s issue, a trend that has previously been observed[24]. Use of modern FP ought to be embraced by all gender and by families rather than individuals because people's perceptions of FP determine whether they will use it or not[32].

Being cross-sectional in design, this study has a limitation in the sense that it cannot be used to establish causality. It should also be noted that the level of knowledge of different methods of FP and sources of information were not assessed. While conducting interviews, we experienced nonresponse or concealment of some FP information by some male respondents; however this was minimal.

## Conclusion

Knowledge of modern FP among people in FCs of Lake Victoria, Uganda is high but its usage is low. Feasible and sustainable strategies of addressing the low usage of modern FP methods in these communities need to be found. High fertility desires, misconceptions about effectiveness of FP, fear of perceived side effects of FP such as excessive bleeding and partner refusal are key barriers to use of modern FP. Community wide promotion of modern FP use through sensitization about its benefits, proven side effects and effectiveness is urgently needed in FCs. Service providers and researchers need to increase access to FP services by bringing the services closer to where people live. Modern FP provision needs to be tailor-made to match the mobile nature, remoteness and high risk behavior of this unique population. Researchers should put in place appropriate mechanisms for providing modern FP methods at research sites, or through referral to quality FP service providers in communities from which volunteers are recruited.
