# Correction: Use of Modern Family Planning Methods in Fishing Communities of Lake Victoria, Uganda

**DOI:** 10.1371/journal.pone.0143988

**Published:** 2015-11-24

**Authors:** Annet Nanvubya, Julius Ssempiira, Juliet Mpendo, Ali Ssetaala, Annet Nalutaaya, Mathias Wambuzi, Paul Kitandwe, Bernard S. Bagaya, Sabrina Welsh, Stephen Asiimwe, Leslie Nielsen, Fredrick Makumbi, Noah Kiwanuka

There are errors in the Funding section. The correct funding statement is as follows: This work was made possible by generous support from many donors including; The training Health Researchers into Vocational Excellence in East Africa Project (THRiVE), Grant Number 087540 of Welcome trust, UK, and the Canada-Africa Prevention Trials Network (CAPTN) Grant Number 1063357–001. It was also funded in part by IAVI and made possible by the generous support of the United States Agency for International Development (USAID) and other donors. The full list of IAVI donors is available at www.iavi.org. The contents of this manuscript are the responsibility of IAVI and co-authors and do not necessarily reflect the views of USAID or the United States Government.
